# WNK1 kinase and the termination factor PCF11 connect nuclear mRNA export with transcription

**DOI:** 10.1101/gad.303677.117

**Published:** 2017-11-01

**Authors:** Adam Volanakis, Kinga Kamieniarz-Gdula, Margarita Schlackow, Nick J. Proudfoot

**Affiliations:** Sir William Dunn School of Pathology, University of Oxford, Oxford OX1 3RE, United Kingdom

**Keywords:** PCF11 CID, WNK1 kinase, mRNA export, transcription termination

## Abstract

Nuclear gene transcription is coordinated with transcript release from the chromatin template and messenger RNA (mRNA) export to the cytoplasm. Here we describe the role of nuclear-localized kinase WNK1 (with no lysine [K] 1) in the mammalian mRNA export pathway even though it was previously established as a critical regulator of ion homeostasis in the cytoplasm. Our data reveal that WNK1 phosphorylates the termination factor PCF11 on its RNA polymerase II (Pol II) C-terminal domain (CTD)-interacting domain (CID). Furthermore, phosphorylation of the PCF11 CID weakens its interaction with Pol II. We predict that WNK1 and the associated phosphorylation of the PCF11 CID act to promote transcript release from chromatin-associated Pol II. This in turn facilitates mRNA export to the cytoplasm.

## Abstract (precis)

In this study, Volankis et al. present evidence for a new connection between gene transcription and mRNA export. They show that the kinase WNK1 phosphorylates termination factor PCF11 on its RNA polymerase II CTD-interacting domain (CID) and suggest that WNK1 and the associated phosphorylation of the PCF11 CID act to promote transcript release from chromatin-associated Pol II, which facilitates mRNA export to the cytoplasm.

Gene expression is a multistep and highly regulated process. It begins with transcription of a gene in the nucleus and ends with translation of the messenger RNA (mRNA) in the cytoplasm (Moore and Proudfoot 2009). After transcription initiation, RNA polymerase II (Pol II) enters the elongation phase, during which time nascent RNA is processed (5′ cap and splicing) and several protein complexes assemble on the pre-mRNA, ultimately leading to formation of the final mRNA protein (mRNP) complex. 3′ end processing of the nascent RNA then releases polyadenylated mRNA from the chromatin template and at the same time triggers Pol II termination.

As Pol II transcribes a gene, the C-terminal domain (CTD) of its largest subunit, which comprises the heptadic repeat structure YSPTSPS (repeated 52 times in the mammalian enzyme, 26 times in yeast), is progressively phosphorylated, in particular at S2 positions (for reviews, see Milligan et al. 2016; Zaborowska et al. 2016). This phosphorylation mark is then recognized by the cleavage and polyadenylation complex (CPAC), subsequently leading to 3′ end processing of the pre-mRNA. More specifically, CID (CTD-interacting domain)-containing factors such as PCF11 recognize and associate with the modified CTD of Pol II (Hsin and Manley 2012). When Pol II transcribes past the pA site (PAS), it pauses, and the transcript is cleaved by CPSF73. This is then polyadenylated, and the released mRNA is engaged in the process of nuclear export. Transcript cleavage also generates a 5′ end on the downstream nascent RNA, which provides an entry point for the exonuclease XRN2 (Rat1 in yeast) to degrade this transcript (West et al. 2004, 2008; Luo et al. 2006; West and Proudfoot 2008). XRN2 potentially acts like a “torpedo,” which, in combination with conformational changes in the elongating polymerase, leads to dissociation of Pol II from the DNA template, thereby completing transcription termination (Plant et al. 2005; Kuehner et al. 2011; Porrua and Libri 2015; Proudfoot 2016).

The next step following transcription termination is export of the mRNA out of the nucleus (Rougemaille et al. 2008). This process is initiated cotranscriptionally with the recruitment of export factors and the formation of an export-competent mRNP. In metazoans and *Saccharomyces cerevisiae* (yeast), the export factors UAP56/SUB2 and ALY/YRA1 are cotranscriptionally recruited to the elongation complex through interactions with splicing and transcription factors (Johnson et al. 2009, 2011; Viphakone et al. 2012). This leads to assembly of the transcription export (TREX) complex (Chi et al. 2013), which then marks the mRNP as export-competent following its release from Pol II (for review, see Bjork and Wieslander 2014).

Interestingly, it has been shown that mutation of certain cleavage and polyadenylation (termination) factors (yeast CFIA subunits, RNA14, RNA15, and PCF11) as well as poly(A) polymerase can have inhibitory effects on mRNA export by causing the retention of transcripts in the nucleus. Experiments in yeast have shown that this defect in mRNA export is due to the loss of interaction of the termination factor PCF11 with the export factor YRA1 (ALY in mammals) (Johnson et al. 2009, 2011). Thus, YRA1 is recruited to transcribed loci through its association with PCF11, a core termination factor (Amrani et al. 1997; West and Proudfoot 2008) that is required for the correct assembly of an export-competent mRNP. Defects in mRNA export lead to the accumulation of termination factors at the 3′ ends of genes. This suggests that mRNA export and the release of mRNPs from the transcription locus require the coordinated action of export factors such as ALY with 3′ processing factors (Rougemaille et al. 2008). Finally, it has been suggested from studies in yeast that recruitment of the export factor YRA1 to genes through its interaction with PCF11 can modulate transcription termination, possibly by competition with the PCF11 cofactor CLP1 (Johnson et al. 2011).

In the present study, we identify the kinase WNK1 (with no lysine [K] 1) as a factor required for the efficient export of mRNA in human cells. WNK1 is a ubiquitously expressed kinase in higher eukaryotes. WNK proteins constitute a family of serine/threonine protein kinases with an atypical placement of a lysine residue in their catalytic domains that is involved in ATP binding and the catalysis of the phosphate transfer reaction (Xu et al. 2000, 2005a,b). WNK kinases have been shown to be vital regulators of ion homeostasis, particularly in the kidney and nervous system (for review, see Shekarabi et al. 2017). WNK1 is essential for mammalian development and is linked to various human cancers. Furthermore, mutations in the *WNK1* gene cause an autosomal dominant syndrome of hypertension and hyperkalemia (for review, see Rodan and Jenny 2017). WNK1 has been described previously as a primarily cytoplasmic protein (Tu et al. 2011) even though another study indicated that the protein also localizes to the nucleus (Xu et al. 2000). We now show that nuclear WNK1 interacts with termination factor PCF11 and promotes phosphorylation of its CID. WNK1 and the associated PCF11 modification facilitate the release of mRNP from transcription loci and in turn promote the nuclear export of mRNA.

## Results

### WNK1 kinase localizes in the nucleus

**Figure 1.**
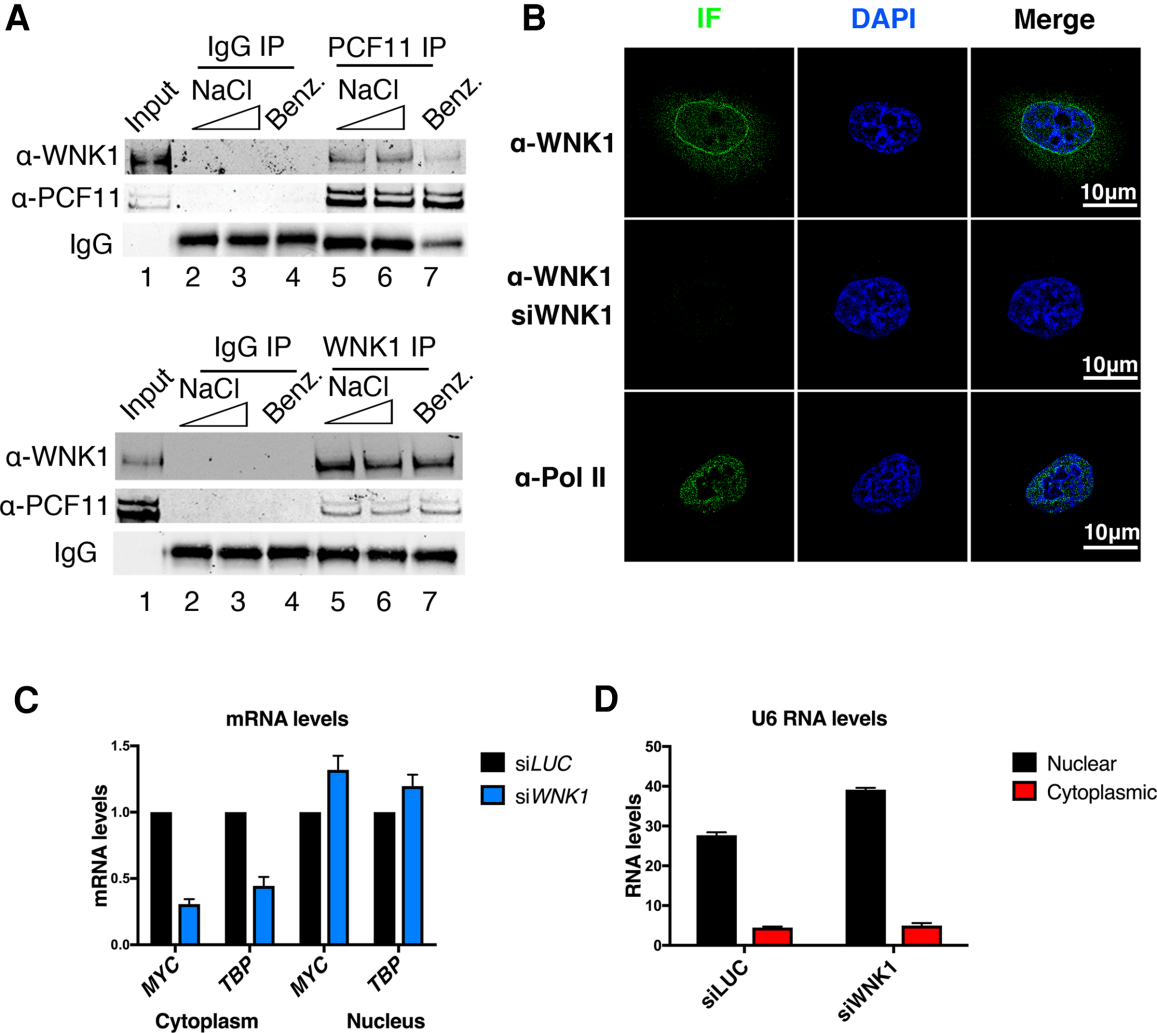
WNK1 is present in the nucleus and interacts with PCF11. (*A*) Co-IP experiments of PCF11 and WNK1. Immunoprecipitation of PCF11 (*top* panel) and WNK1 (*bottom* panel). Lane *1* corresponds to 5% input. Lanes *2*–*4* show mock immunoprecipitation using rabbit IgG (negative control). Lanes *2* and *5* show immunoprecipitation with 150 mM NaCl, and lanes *3* and *6* show immunoprecipitation with 250 mM NaCl. Lane *7* shows immunoprecipitation with 150 Mm NaCl after incubation with benzonase to test for nucleic acid dependence. (*B*) Superresolution microscopy and detection of WNK1 kinase. Pol II staining was used as a control for nuclear localization. The same pattern was observed in all imaged cells. *n* = 20. Data are from two biological repeats. The same laser intensity was used for all images. (*C*) Cytoplasmic and nuclear levels of *MYC* and *TBP* mRNA in control and WNK1-depleted cells. Data are from biological repeats. Error bars represent standard error of mean (SEM). Data were normalized to siRNA targeting luciferase (siLUC). (*D*) U6 RNA levels measured with RT-qPCR in nuclear and cytoplasmic extracts.

Analysis of PCF11-interacting proteins in HeLa cells by mass spectrometry (MS) unexpectedly identified the kinase WNK1 (false discovery rate [FDR] = 0.001; 11 unique peptides) (Supplemental Fig. S1A–C). We verified this interaction by reciprocal coimmunoprecipitation (co-IP) experiments (Fig. 1A) and found a low-level but reproducible interaction of WNK1 with PCF11. Since our co-IP experiments were performed with nuclear extracts, this suggests the nuclear presence of WNK1 even though it was previously described as a cytoplasmic protein. To further verify this result, we used immunostaining to investigate the localization of the kinase. Our data indicate (Fig. 1B) that WNK1 is present in both the cytoplasm and nucleus. Notably, we observed a concentration of WNK1 in a perinuclear location. We also performed nuclear and cytoplasmic fractionation followed by Western blot and detected WNK1 in both fractions (Supplemental Fig. S1D). *WNK1* is 32-exon gene, of which eight can be alternatively spliced in a tissue-specific manner, resulting in multiple protein isoforms (Supplemental Fig. S2; Vidal-Petiot et al. 2012). It is possible that the antibodies and methods used in previous studies detected different isoforms with varying efficiency. The epitope of the antibody used in our study overlaps with a predicted nuclear localization signal (NLS). It is therefore likely that the isoforms detected have the capacity to enter the nucleus (Supplemental Fig. S2).

### WNK1 is required for mRNA export

The observed perinuclear localization of WNK1 suggests a possible connection with nuclear export. We therefore measured the effects of WNK1 depletion by siRNA treatment (Supplemental Fig. S3A) on nuclear and cytoplasmic mRNA levels. Two genes, *MYC* and *TBP*, were tested for nuclear and cytoplasmic mRNA expression levels by RT-qPCR. Notably, we detected an increase in mRNA levels in the nuclear fraction and concomitant reduction in the cytoplasm following WNK1 depletion (Fig. 1C). As a control for the fractionation, we verified that U6 snRNA remained nuclear-specific following siRNA treatment (Fig. 1D). While the increase in nuclear mRNA levels appears modest, this can be attributed to the fact that accumulation of nuclear mRNA can trigger degradation by the nuclear surveillance machinery. These data support a role for WNK1 in mRNA export. To control for siRNA-specific effects, two independent siRNA treatments were used for this experiment: a pool of four siRNAs and the independent single siRNA. Both treatments had similar results on mRNA export (Supplemental Fig. S3B).

**Figure 2.**
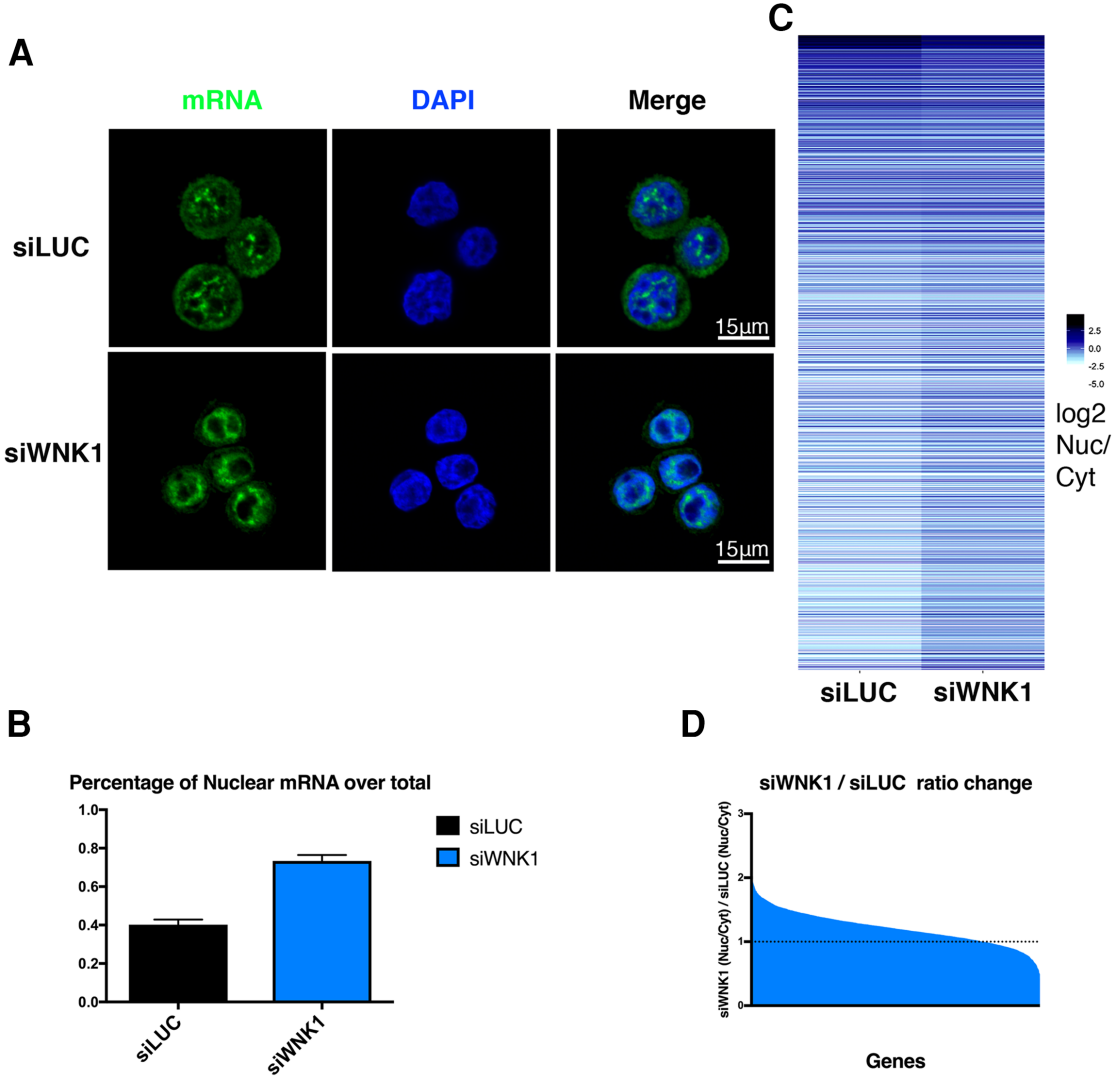
WNK1 participates in mRNA export. (A) Fluorescent oligo-dT FISH for the detection of mRNA levels in control and WNK1-depleted cells. Alexa fluor 488-dT_(23)_ was used as a probe, and DAPI was used for DNA staining. (*B*) Quantitation of the percentage of nuclear-localized mRNA over total. The integrated intensity of the nuclear fluorescent signal and the total fluorescent signal were calculated for every cell (*n* = 30; data are from two biological repeats), and the ratio between the two is presented graphically. Error bars represent SEM. (*C*) Heat map representing the log_2_ ratio of nuclear over cytoplasmic (Nuc/Cyt) mRNA in control and WNK1-depleted cells for the top 20% of expressed genes. The darker shade indicates an increased Nuc/Cyt ratio. (*D*) Quantification of the fold change of Nuc/Cyt mRNA levels between siLUC and siWNK1 cells in the top 20% of expressed genes. Most genes in this set show an increase of Nuc/Cyt ratio upon WNK1 depletion.

To generalize the above results, we performed mRNA FISH using a fluorescent oligo-dT probe [T_(23)_; Alexa fluor 488]. HeLa cells were transfected with the control or WNK1-specific siRNA for 72 h and stained with this fluorescent probe. In the control cells transfected with siRNA targeting luciferase (siLUC), polyadenylated mRNA was equally distributed between cytoplasmic and nuclear compartments (Fig. 2A). However, for cells depleted in WNK1, we observed a clear decrease in cytoplasmic signal and a modest increase in the nuclear signal. Quantitation of the fluorescent signal between the nucleus and cytoplasm showed that there is an almost twofold increase in the ratio of nuclear/cytoplasmic mRNA upon WNK1 depletion (Fig. 2B). This experiment indicates that WNK1 plays a global role in mRNA export in HeLa cells.

To further test this hypothesis and investigate whether there is any specificity in the genes whose export is affected by WNK1, we performed RNA sequencing (RNA-seq) analysis. Control and siRNA WNK1-depleted HeLa cells were fractionated into nuclear and cytoplasmic mRNA preparations and used for library preparation. We calculated the ratios of nuclear over cytoplasmic (Nuc/Cyt) levels of mRNA for the top 20% of expressed genes (∼2600 genes) (Fig. 2C). The ratio is represented as a heat map in control and WNK1-depleted cells, with a darker shade corresponding to an increase in the Nuc/Cyt ratio. The majority of the genes analyzed shows an increase of the Nuc/Cyt mRNA levels upon WNK1 depletion (Fig. 2C,D).

Finally, we performed a gene ontology (GO) analysis of the most highly affected genes (Nuc/Cyt ratio change of >1.5) to test whether WNK1 affects the mRNA export of a specific set of genes (Supplemental Fig. S3C; Supplemental Table 1). The GO analysis did not reveal any enrichment of specific sets of genes, suggesting that WNK1 participates in the general mRNA export mechanism.

### WNK1 is required for the release of mRNP from chromatin

To understand how WNK1 participates in mRNA export, we tested whether its depletion could impair the recruitment of export factors. These are recruited early during the transcription cycle, and a major component of the export machinery is the factor ALY (YRA1 in *S. cerevisiae*). In yeast, YRA1 is recruited to the transcribed gene through its interaction with the termination factor PCF11 (Johnson et al. 2009). Furthermore, YRA1 recruitment is thought to provide cross-talk between 3′ end formation and mRNA export, since it competes with the termination cofactor CLP1 (Johnson et al. 2011). Chromatin immunoprecipitation (ChIP) experiments were performed on WNK1-depleted HeLa cells to test recruitment of ALY to *MYC* and *ACTB* gene loci. However, no significant change in ALY recruitment was evident at these gene loci (Supplemental Fig. S4A).

**Figure 3.**
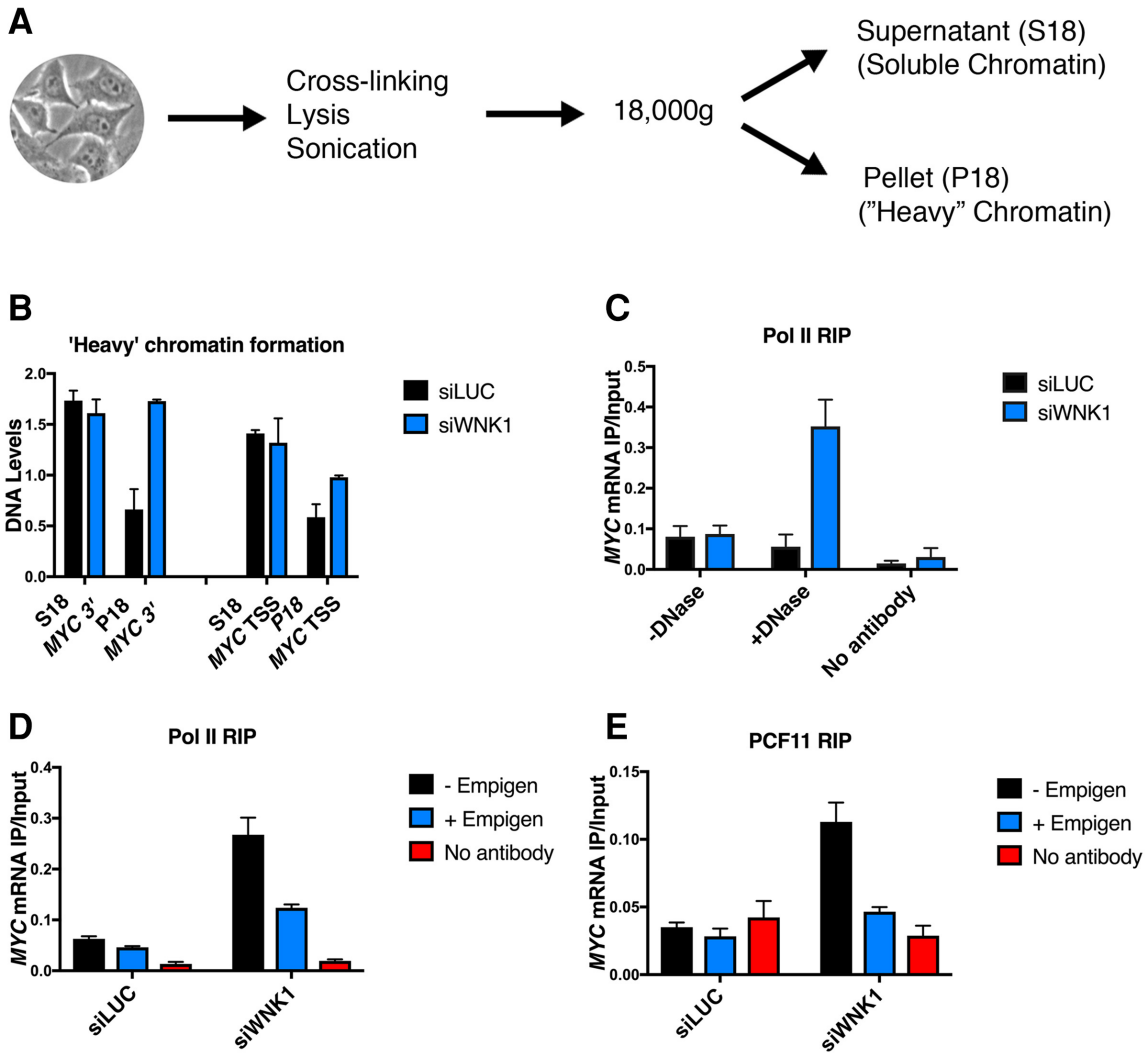
WNK1 is required for mRNA release from the transcription locus. (*A*) Schematic representation of the procedure for the detection of heavy chromatin. (*B*) Detection of the *MYC* transcription start site (TSS) and 3′ DNA in the heavy (P18) and light (S18) fractions in control cells and WNK1-depleted cells. *MYC* DNA levels were measured by qPCR. Results were normalized to levels of a gene desert region. (*C*) Levels of *MYC* mRNA (measured by RT-qPCR) associated with immunoprecipitated RNA Pol II with or without DNase I treatment. Levels were measured in control cells (siLUC) and WNK1-depleted cells (siWNK1). (*D*) Levels of *MYC* mRNA associated with immunoprecipitated Pol II from DNase-treated extracts in control and WNK1-depleted cells with or without empigen treatment. (*E*) Levels of *MYC* mRNA associated with immunoprecipitated PCF11 from DNase-treated extracts in control and WNK1-depleted cells with or without empigen treatment. (*B*–*E*) Data are from three biological replicates. Error bars represent SEM.

As a further test for the participation of WNK1 in mRNA export, a previously described method for the detection of “heavy” chromatin was used (Rougemaille et al. 2008; Jensen et al. 2013). Deletion or mutation of a protein participating in transcription and mRNA export can lead to the stalling of the transcription process at the coupled termination and nuclear export stages of gene expression. This can be detected through the formation of insoluble chromatin retained in the pellet fraction after cell lysis and sonication (Fig. 3A). Using either control (siLUC) or WNK1-depleted cells and qPCR for the detection of *MYC*-associated chromatin, the majority was detected in the supernatant fraction (S18). However, in WNK1 knockdown samples, an increase in *MYC* chromatin levels was observed in the pellet fraction (P18) (Fig. 3B), especially on the MYC 3′ end region. These results indicate that WNK1 depletion impairs the release of mRNP from the transcription locus, resulting in the generation of “heavy” chromatin.

As an alternative approach to investigate this “heavy” chromatin formation following WNK1 depletion, we measured the levels of *MYC* mRNA that remains Pol II-associated rather than released from the chromatin template by an RNA immunoprecipitation assay (RIP). Cellular extracts were prepared with or without DNase I treatment, with the former acting to release Pol II from insoluble chromatin. RIP using Pol II antibodies allowed the isolation of solubilized Pol II-associated mRNA from these extracts. Reverse transcription with oligo-dT primer was used to ensure that we detected only mature mRNA. In control cells, both DNase-treated (representing total Pol II) and DNase-untreated (nucleoplasmic Pol II) extracts showed only close to background interaction with the test *MYC* mRNA. This suggests that most mature *MYC* mRNA is released from Pol II. In contrast, WNK1 depletion resulted in a fivefold increase in *MYC* mRNA levels associated with Pol II from the DNase I-treated extracts, implying increased *MYC* mRNA retention on its chromatin template (Fig. 3C). This result strengthens the above “heavy” chromatin results, arguing that WNK1 depletion causes a failure of mRNA release from chromatin. To further test whether this Pol II-associated mRNA is attached to the active site of Pol II, we repeated this experiment but using pretreatment with empigen. This strong detergent acts to disrupt RNA–protein and protein–protein interactions. However, protected interactions such as RNA inside the active site of Pol II will be unaffected (Choi and Dreyfuss 1984; Nojima et al. 2016). We again detected mRNA associated with the polymerase using the DNase I-treated extracts, but this interaction is greatly reduced following empigen treatment (Fig. 3D). This result suggests that the interaction of mRNA with Pol II is readily dissociable and possibly occurs through other protein contacts. To test whether termination factors are also associated with the mRNA on the chromatin, we repeated this RIP analysis by immunoprecipitation of PCF11 (Fig. 3E). Similarly to Pol II, upon WNK1 depletion, mRNA was found in association with PCF11, and, again, empigen treatment greatly reduced this interaction. The above results all point to a role of WNK1 kinase in the release of mRNA from the transcribed locus. Depletion of WNK1 leads to the accumulation of mRNA associated with Pol II and termination factors. We interpret these results as a failure of mRNP to release from the transcription locus following WNK1 depletion. To corroborate these data, the same experiment was repeated for two more genes (*TBP* and *SGK1*), and, again, WNK1 depletion had a similar effect (Supplemental Fig. S4B). Overall, our immunoprecipitation experiments (Fig. 3) indicate that WNK1 affects mRNA export by promoting release of mRNP from the transcription locus. Thus, depletion of WNK1 leads to the accumulation of mRNA at transcription loci through its interaction with transcription factors and the polymerase.

### WNK1 phosphorylates the PCF11 CID

**Figure 4.**
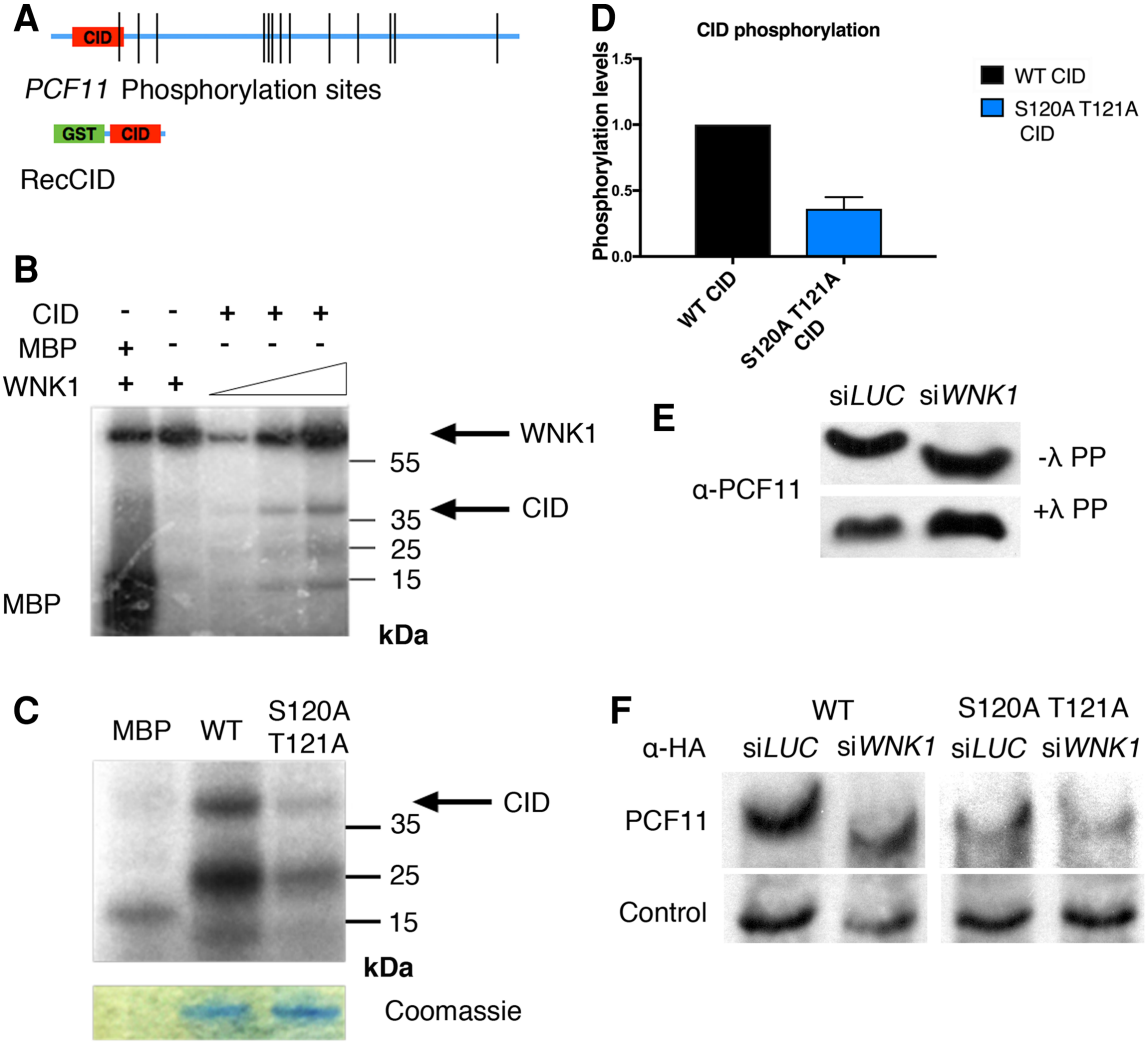
WNK1 phosphorylates the PCF11 CID. (*A*, *top*) Schematic representation of endogenous PCF11 phosphorylation sites identified in this study (Supplemental Table 2). (*Bottom*) Schematic representation of the recombinant GST-PCF11 CID. (*B*) Autoradiography of a recombinant WNK1 kinase domain in vitro kinase assay on the GST-PCF11 CID. MBP was used as a positive control for WNK1 phosphorylation. The upper band corresponds to autophosphorylated WNK1. The lower bands likely correspond to truncated products of the GST-PCF11 CID. (*C*) Autoradiography of a WNK1 in vitro kinase assay on the wild-type CID and S120A–T121A CID. Coomassie staining shows equal levels of GST-CID loading for wild type and the mutant. (*D*) Quantitation of kinase assays with the wild-type and S120A–T121A CID. Phosphorylation levels were measured in four independent repeats of the assay. Error bars represent SEM. (*E*) PhosTag Western blot for the detection of endogenous PCF11 migration upon WNK1 depletion. The same cell extracts treated with λ phosphatase were used as controls. (*F*) PhosTag Western blots comparing migration of wild-type PCF11-HA and S120A–T121A PCF11-HA in LUC and WNK1-depleted cells. The control lane shows the migration of an unspecific band recognized by the αHA antibody, which provides a positional and loading control.

Since WNK1 is a protein kinase and we observed its weak interaction with PCF11 (Fig. 1A), we tested whether WNK1 can phosphorylate PCF11. First, we performed in silico kinase-specific phosphorylation site prediction on PCF11 using the GPS 2.1 method (Xue et al. 2008). Interestingly, the WNK kinase family is predicted to phosphorylate a unique site in PCF11: Thr121 (T121) in the CID of PCF11. However, the PCF11 CID has not been reported to be phosphorylated in vivo in either the literature or protein databases. Therefore, we performed a targeted MS experiment on purified endogenous PCF11 protein. This allowed us to detect phosphorylation of the CID as well as 11 other phosphorylation sites on PCF11 (Fig. 4A; Supplemental Fig. S5; Supplemental Table 2). Unfortunately, MS analysis was unable to distinguish between phosphorylation of the CID T121 or neighboring S120 residues. Because we were not able to determine which of the two residues is modified in vivo, we used the abbreviation S120/T121 to indicate S120 and/or T121.

To determine whether WNK1 is, as predicted, able to phosphorylate the PCF11 CID, we expressed and purified the recombinant PCF11 CID from bacteria using an N-terminal GST tag (Fig. 4A) and performed in vitro kinase assays with a recombinant WNK1 kinase domain. Following a 10-min reaction with ^32^Pγ-ATP, labeled products were separated on acrylamide gels (Fig. 4B). WNK1 phosphorylates the positive substrate MBP and autophosphorylates itself, which is required for its kinase activity (Xu et al. 2000; Meinhart and Cramer 2004). Importantly, WNK1 phosphorylates the PCF11 CID in a dosage-dependent manner (Fig. 4B). Since this approach does not distinguish which residues on the CID are phosphorylated, we repeated the same assay using a CID protein bearing a double S120A and T121A mutation (Fig. 4C,D). Notably, the phosphorylation levels of the S120A–T121A mutant dropped by >50%, suggesting that the detected phosphorylation is specific for S120/T121. Note that equal amounts of GST-CID were added for wild-type and mutant protein based on Coomassie staining. As a further verification, we subjected the band corresponding to the wild-type PCF11 CID after incubation with WNK1 to MS. This assay confirmed the phosphorylation of either S120 or T121 (Supplemental Fig. S6; Supplemental Table 3). None of the 10 other serine and threonine residues in the PCF11 CID that were within the MS coverage showed any detectable phosphorylation. Those experiments establish that the WNK1 kinase domain has the capacity to phosphorylate PCF11 S120/T121 in vitro.

To determine whether WNK1 depletion affects PCF11 phosphorylation in vivo, we used the PhosTag reagent*.* PhosTag is a compound that interacts with phosphorylated residues of proteins, causing slower protein migration on gels (Kinoshita et al. 2006). We first assayed the migration of endogenous PCF11 in a PhosTag gel and found that upon WNK1 depletion, PCF11 migrates faster than in control cells (Fig. 4E), implying decreased phosphorylation. The same samples treated with λ phosphatase migrate at the same position. This indicates that WNK1 phosphorylates PCF11 in vivo*.* To further test whether the observed PCF11 phosphorylation is specific to S120/T121, we again compared the migration of ectopically expressed HA-tagged wild-type and S120A–T121A PCF11 with or without WNK1 depletion on a PhosTag gel (Fig. 4F). Upon WNK1 depletion, wild-type PCF11 yielded a faster-migrating band as compared with the unchanged control band. This result is consistent with the endogenous PCF11 migration data (Fig. 4E). In contrast, WNK1 depletion had no effect on S120A–T121A PCF11 band migration (Fig. 4F). This strongly suggests that S120/T121 is a WNK1 kinase target site. Overall, both our in vitro and in vivo data indicate that PCF11 CID S120 and/or T121 is a target for phosphorylation by the WNK1 kinase.

### PCF11 CID phosphorylation mediates mRNA export

The above results (Figs. 1–3) show that WNK1 plays a critical role in mRNP release from transcription sites and subsequent nuclear export of mRNA. Furthermore, WNK1 can phosphorylate the PCF11 CID both in vitro and in vivo (Fig. 4). We therefore sought to connect these two observations by testing whether phosphorylation of the PCF11 CID also affects mRNA export.

**Figure 5.**
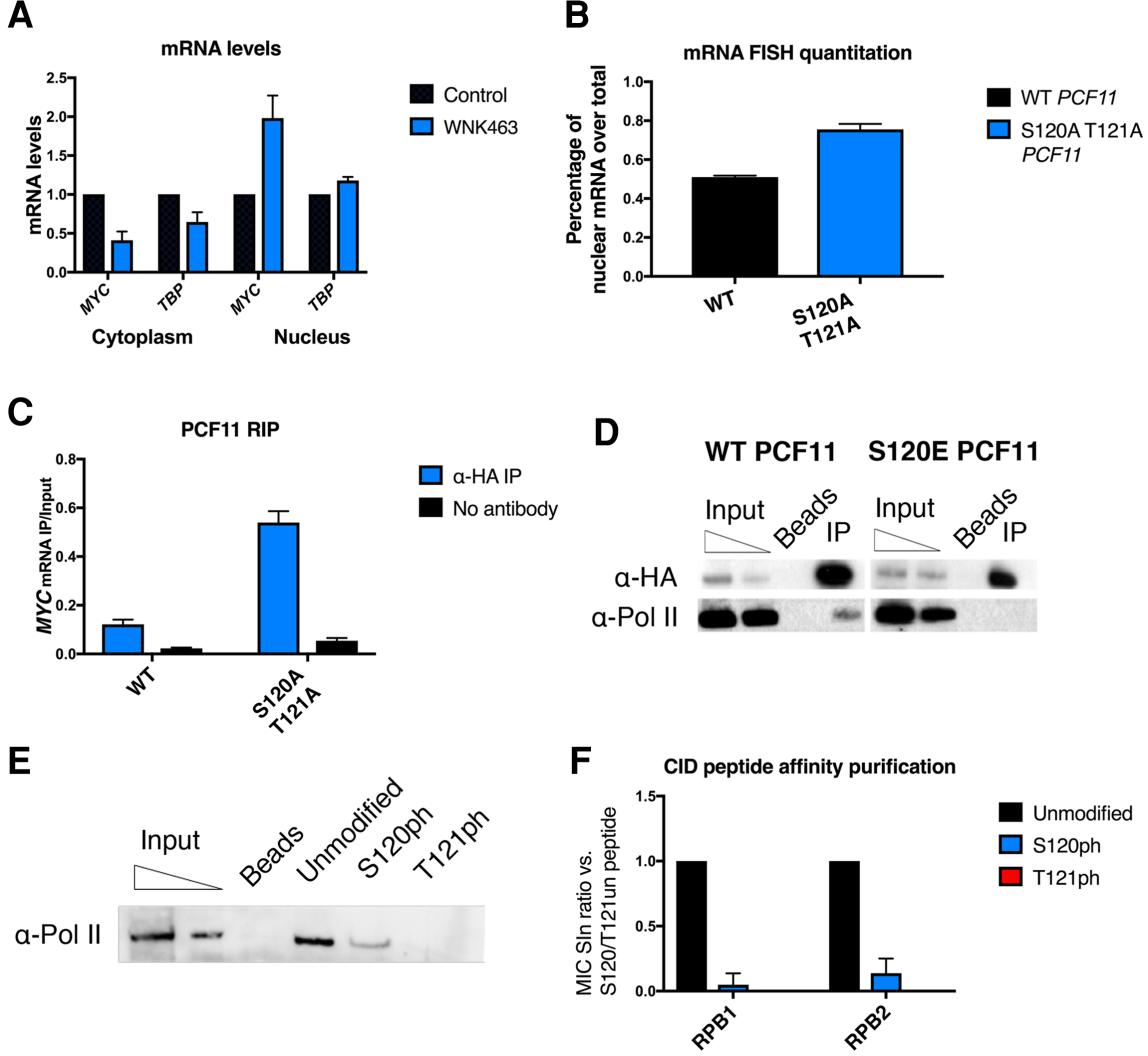
Effect of PCF11 CID phosphorylation on mRNA export and Pol II interaction. (*A*) Cytoplasmic and nuclear levels of *MYC* and *TBP* mRNA upon WNK463 treatment for 6 h. Data are from two biological repeats. Error bars represent SEM. Data were normalized to siLUC. (*B*) Quantitation of the percentage of nuclear-localized mRNA over total in fluorescent oligo-dT FISH in cells expressing wild-type or S120A–T121A PCF11 (Supplemental Fig. 7C). The integrated intensities of the nuclear fluorescent signal and the total fluorescent signal were calculated. *n* = 15. The ratio between the two is presented graphically. Summary data of two biological repeats are shown. Error bars represent SEM. Only cells ectopically expressing PCF11-HA were measured. (*C*) Detection of *MYC* mRNA associated with immunoprecipitated wild-type and S120A/T121A PCF11. (*D*) Co-IP experiment of PCF11 and RNA Pol II. PCF11 immunoprecipitation was followed by Western blot for the detection of Pol II–PCF11 interaction and the comparison of ectopically expressed wild-type and phosphomimetic S120E. (*E*) RNA Pol II binding to PCF11 CID peptides—unmodified (un) or phosphorylated on S120 (S120ph) or T121 (T121ph)—assayed by Western blot. The first two bands correspond to 5% and 1% of input. A representative blot from three independent experiments is shown. (*F*) MS analysis of peptide affinity-purified proteins binding to PCF11 CID peptides. The ratio of the spectral index (MIC SIn) of the indicated peptide versus unmodified S120/T121 peptide is shown. Unmodified peptides bind both RPB1 and RPB2 subunits of Pol II in MS, whereas Pol II binding to the same peptide phosphorylated on S120 is ∼10-fold lower, and Pol II binding to the peptide phosphorylated on T121 is entirely lost. Error bars represent standard deviation in three independent experiments. Full data are available in Supplemental Table 3.

First, we tested whether WNK kinase activity and not merely WNK1 presence is required for mRNA export by use of a pan-WNK kinase inhibitor called WNK463 (Yamada et al. 2016). We confirmed WNK463 inhibition of WNK1 activity by showing that phosphorylation of a known WNK1 target, S325p of OSR1, was blocked by WNK463 treatment (Supplemental Fig. S7A). However WNK463 treatment had no noticeable effects on WNK1 protein levels (Supplemental Fig. S7B). To test whether the enzymatic activity of WNK1 is required for mRNA export, we treated the cells with WNK463 for 6 h, after which we performed fractionations of nuclear and cytoplasmic mRNA. WNK463 treatment leads to the decrease of cytoplasmic mRNA levels and an increase of nuclear mRNA levels of *MYC* and *TBP* (Fig. 5A) similar to those observed upon WNK1 depletion. This result confirms that WNK kinase activity is required for mRNA export.

Our experiments have shown that PCF11 CID S120/T121 can be phosphorylated by WNK1. To test the effect of phosphorylation on these residues in mRNA export, we ectopically expressed C-terminally HA-tagged PCF11 in HeLa cells as either wild type or with a double S120A–T121A mutation mimicking unphosphorylated protein. mRNA FISH analysis was performed using the fluorescent oligo-dT probe 48 h after transfection. Cells expressing wild-type PCF11 showed a normal distribution of mRNA in the nucleus and cytoplasm. However, expression of S120A–T121A PCF11 revealed a change in mRNA distribution (Supplemental Fig. S7C) similar to the mRNA export defect observed in WNK1-depleted cells (Fig. 2A). Quantitation of the fluorescent signal again revealed an increase in the ratio of Nuc/Cyt mRNA, suggestive of an mRNA export defect (Fig. 5B). It is notable that the export defect observed for transfected S120A–T121A PCF11 is not as severe as with WNK1 depletion. However, in this experiment, cells also express endogenous wild-type PCF11. It appears that expression of the S120A–T121A PCF11 acts as a dominant-negative mutant.

We next tested whether CID phosphorylation could affect the interaction of export factors with PCF11. To this end, we performed co-IP experiments of PCF11 with ALY and NPC (nuclear pore complex). While both ALY and NPC were detectable upon immunoprecipitation of PCF11, we did not observe any significant change in these interactions when a phosphomimetic PCF11 S120E mutant was used (Supplemental Fig. S8A). This suggests that the mechanism by which both WNK1 and PCF11 CID phosphorylation impact mRNA export is likely independent of export factor recruitment.

Since WNK1 loss affected mRNP release, we wanted to check whether PCF11 CID modification could have a similar effect. We therefore performed RIP experiments using ectopic HA-tagged PCF11 either wild type or S120A–T121A mutant. As with endogenous PCF11 (Fig. 3E), wild-type PCF11-HA associated only with background levels of *MYC* mRNA in DNase-treated extracts. In contrast, the S120A–T121A mutant showed binding of substantial levels of *MYC* mRNA (Fig. 5C). These results imply that S120/T121 phosphorylation is required for mRNP release from the chromatin.

### PCF11 CID phosphorylation weakens its interaction with RNA Pol II

We finally addressed the mechanism by which phosphorylation of the PCF11 CID could affect mRNP release and subsequent mRNA export. The PCF11 CID is a highly conserved domain responsible for interaction with the CTD of RNA Pol II. We used the published structure of the yeast PCF11 CID in complex with a Pol II CTD peptide (Meinhart and Cramer 2004) to make predictions of what impact S120 and T121 phosphorylation could have on the CTD interaction. T121 corresponds to one of the residues directly contacting the CTD, and its phosphorylation would predictably disturb the hydrophobic contact site. In contrast, the S120 position on the α helix directs it away from the contact site, so the influence that a phosphate group would have in this location is unclear. In order to test the effect of PCF11 phosphorylation on its interaction with Pol II, we performed co-IP experiments using ectopically expressed HA-tagged wild-type PCF11 and the phosphomimetic S120E mutant. Notably, the interaction of Pol II with wild-type PCF11 was readily visible, whereas it was undetectable with the S120E mutant (Fig. 5D). This result suggests that modification of S120 is able to disrupt the interaction of the CID with the Pol II CTD. Since a phosphomimetic mutation is not identical to phosphorylation, we next performed peptide affinity purification experiments using synthetic PCF11 CID peptides either unmodified or phosphorylated on S120 or T121 (Supplemental Table 5). Affinity purification of nuclear extract proteins interacting with the CID peptides was followed by Western blot and MS to detect RNA Pol II (Fig. 5E,F; Supplemental Fig. S8B; Supplemental Table 4). While the unmodified CID peptide interacts with Pol II, this was substantially weakened with the S120 phosphorylated peptide and abolished with the T121 phosphorylated peptide.

These experiments reveal that phosphorylation of either S120 or T121 has the potential to disrupt the interaction of the Pol II CTD with the CID of PCF11. We predict that phosphorylation of S120/T121 by WNK1 promotes the dissociation of PCF11 from Pol II. This will act to promote the release of PCF11, Pol II, and the mRNP from the transcription site.

## Discussion

Here we identified a new role for the kinase WNK1 in mRNA export. Our results reveal that WNK1 localization is not limited to the cytoplasm, as suggested previously, but also shows a strong nuclear presence (Fig. 1; Supplemental Fig. S1D). We suspect that different isoforms of WNK1 may account for why the nuclear location of this kinase was not appreciated previously. Our results indicate that both WNK1 presence and kinase activity are required for mRNA export, as its knockdown and treatment with the WNK463 inhibitor lead to mRNA retention in the nucleus (Figs. 1C, 2, 5A). Our data also suggest that WNK1 participates in the mRNA export pathway by directing the release of mRNP from Pol II and transcription factors at transcription loci (Fig. 3).

To further elucidate the mechanism of action of WNK1, we identified PCF11 as a likely substrate for the kinase. We show that PCF11 and WNK1 interact in nuclear extracts (Fig. 1A; Supplemental Fig. S1). In silico prediction suggested T121 as a unique residue in PCF11 that could be a substrate for WNK family kinases. Furthermore, our in vitro experiments confirmed that the recombinant WNK1 kinase domain is capable of phosphorylating S120/T121 in the PCF11 CID (Fig. 4). Even though PCF11 CID phosphorylation has not been detected previously in vivo, we were able to confirm S120/T121 phosphorylation on endogenous PCF11 by targeted MS (Supplemental Fig. S5; Supplemental Table 2). We further used a PhosTag approach and found that WNK1 promotes wild-type PCF11 phosphorylation but not the S120A–T121A mutant, suggesting that WNK1 is required for phosphorylation of PCF11 CID S120/T121 in vivo. Consistent with WNK1 kinase activity acting on PCF11 S120/T121, the effects of WNK1 depletion and PCF11 S120/T121 mutations phenocopy each other. Thus, ectopic expression of S120A–T121A results in the retention of mRNAs in the nucleus and in prolonged association of PCF11 with polyadenylated mRNAs at transcription loci (Fig. 5B,C; Supplemental Fig. S7C).

Interestingly, phosphorylated or phosphomimetic S120 and T121 disrupt the interaction of PCF11 with RNA Pol II (Fig. 5D–F). We predict that phosphorylation of S120/T121 by WNK1 is a necessary step for the disruption of the CID–CTD interaction after transcription termination. This will promote the dissociation of mRNP from the transcription locus and so lead to its efficient export.

Multiple lines of evidence (see above) support WNK1 as a PCF11 S120/T121 kinase. Nevertheless, it is possible that additional kinases may also be able to phosphorylate the PCF11 CID in certain circumstances. For example, this has been shown for Pol II CTD residues, which are often phosphorylated by multiple kinases (Zaborowska et al. 2016). We also note that it is likely that WNK1 has additional substrates and potentially other roles in the nucleus, which will be interesting to investigate further.

**Figure 6.**
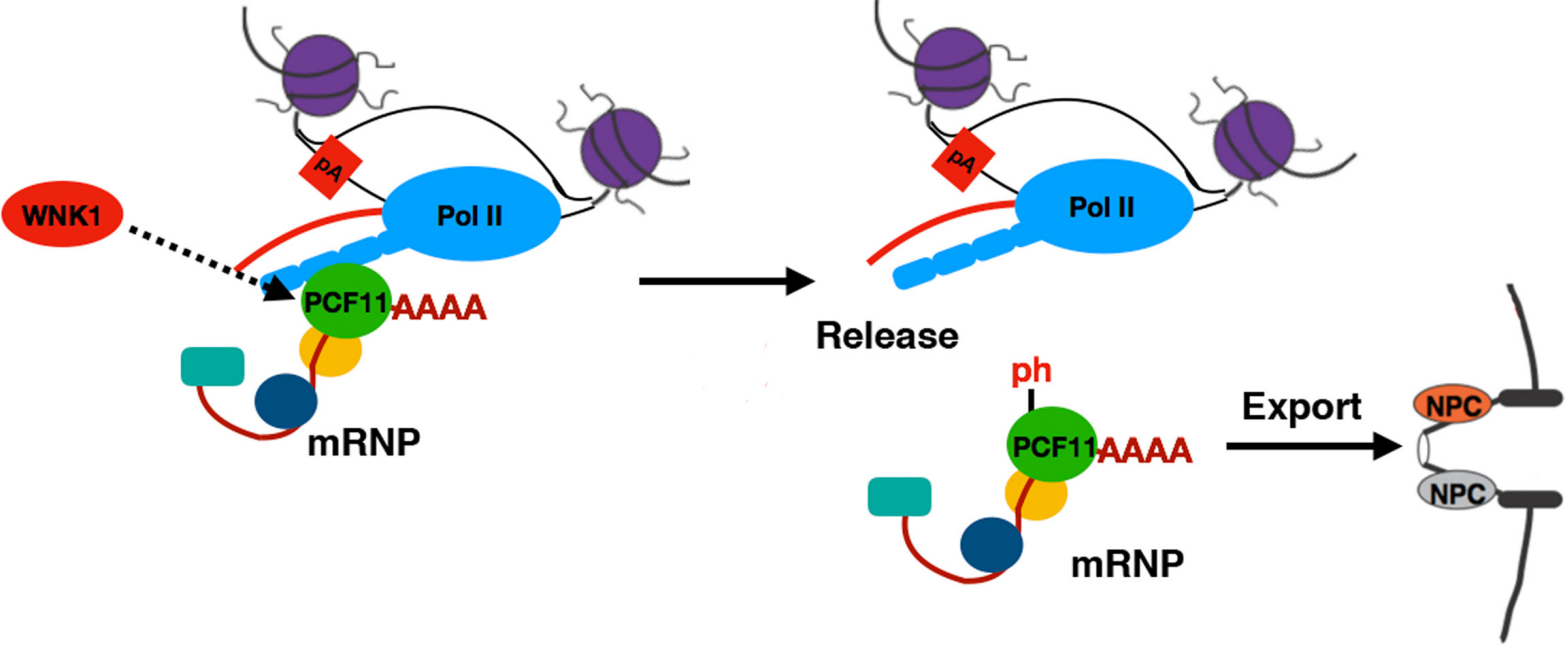
Model. Following cleavage and polyadenylation of the transcript, the CID of PCF11 is transiently phosphorylated by WNK1. This weakens the interaction between the PCF11 CID and the Pol II CTD, leading to the dissociation and release of mRNP from transcription loci. As a result, nuclear export of mRNA is promoted.

Overall, our study provides insight into a new layer of mRNA export regulation through phosphorylation of the PCF11 CID and identifies the role of the WNK1 kinase in this process. PCF11 CID S120/T121 phosphorylation by WNK1 promotes the disassembly of the transcription termination complex and release of the mRNP from the transcription locus. Our results point toward a model (Fig. 6) in which, following cleavage and polyadenylation of the transcript, the CID of PCF11 is transiently phosphorylated by WNK1. Such phosphorylation will act to weaken the interaction between the PCF11 CID and the Pol II CTD. This will then lead to the essential dissociation and release of mRNP from transcription loci and so promote the nuclear export of mRNA.

## Materials and methods

### Cell culture and siRNA treatment

HeLa cells were used for all experiments in this study. Cell culture was performed as described previously (Skourti-Stathaki et al. 2014). siRNA treatment was performed using Lipofectamine RNAimax (Thermo, 13778030) as described in the product manual. For WNK1 depletion, cells were harvested 48 h after transfection with 10 nM siRNA. The efficiency of depletion was tested by Western blot with the appropriate antibodies.

### Antibodies

The antibodies used were as follows: Pol II (Santa Cruz Biotechnology, SC-816), WNK1 (Bethyl Laboratories, A301-514A), NPC (Abcam, ab24609), ALY (Abcam, ab6141), HA (Abcam, ab9110), PCF11 (Abcam, ab134391), pS326 OSR1 (Merck, 07-2273), and rabbit IgG control (Abcam, ab37415). Note that the performance of the polyclonal PCF11 antibody (Abcam, ab134391) displayed strong lot dependency. In our assays lots, GR101530-1 and GR101530-2 had the capacity to immunoprecipitate CID phosphorylated PCF11, whereas lots GR101530-4 and GR250552-3 did not. In Figure 1A, IgG was visualized by secondary antibody alone (anti-rabbit; Li-COR, 926-32211).

### In vitro kinase assay

The PCF11 CID with an N-terminal GST tag was expressed from the pGEX-4T1 tag vector in BL21 *Escherichia coli* cells. Expression was induced with 10 mM IPTG for 3 h at 25°C. The GST-CID was purified using glutathione columns (Pierce glutathione cartridge, 16110), subsequently applied to a Pierce protein concentrator column (PES, 10-k molecular weight cutoff [MWCO], CN88517), and centrifuged at 15,000*g* for 15 min. For this assay, a commercially available recombinant WNK1 catalytic domain was used (ProQinase, CN 1111-0000-11) with the recombinant PCF11 CID. One microgram of recombinant WNK1 and 5 µg of the recombinant CID were incubated in a kinase reaction buffer (100 mM HEPES at pH 7.9, 100 mM MgCl_2_, 100 mM MnCl_2_, 1.5 M NaCl, protease, phosphatase inhibitor cocktail) with the addition of 0.2 µL of ^32^P-γATP. For the control reaction, recombinant WNK1 was incubated with 0.5 µg of MBP-positive control substrate in the same buffer and conditions. The samples were incubated for 10 min at 37°C.

### mRNA FISH

Cells were grown in six-well plates on poly-L-lysine-covered coverslips. Forty-eight hours after transfection, coverslips were washed with ice-cold PBS. Cells were then fixed with 4% paraformaldehyde (PFA) in PBS for 10 min at room temperature and incubated in 100% methanol for another 10 min. After fixation, 70% ethanol was added to the cells for 15 min to rehydrate them, followed by a 5-min wash in 100 mM Tris (pH 8).

For probe preparation, 1 ng/µL oligo-dT [T_(23)_] conjugated with Alexa 488 fluorophore was added to hybridization buffer (1 mg/mL yeast tRNA, 0.005% [w/v] BSA, 10% [w/v] dextran sulfate, 25% formamide in 2× SSC buffer) and incubated with cells for 12 h at 37°C. Samples were washed once with 4× SSC followed by two washes for 5 min with 2× SSC. The primary antibody was diluted in 2× SSC and 0.1% Triton and incubated for 1 h at room temperature. The coverslips were subsequently washed three times with 2× SSC and 0.1% Triton. The appropriate dilution of the secondary antibody was added in 2× SSC and 0.1% Triton and incubated for 30 min at room temperature. Standard confocal microscopy was used for imaging (Olympus LiveView), and FIJI software was used for quantitation.

### Heavy chromatin assay

Cells (6 × 10^6^ to 7 × 10^6^) were collected 48 h after transfection. Cells were cross-linked with 1% formaldehyde for 15 min at 37°C, and the cross-linking reaction was stopped with the addition of 125 mM glycine. After nuclear isolation and lysis, the samples were sonicated for 15 min (Bioruptor, maximum setting, 30 sec on/off). Following sonication, the samples were centrifuged at 18,000*g* for 10 min. The pellet was resuspended in lysis buffer 2, and DNA was extracted from both samples using phenol:chloroform followed by qPCR.

### RIP

Cells were lysed in lysis buffer with or without DNase I and lysed on a shaker for 3 h at 4°C. Immunoprecipitation was performed overnight on a rotator at 4°C using an antibody against RNA Pol II (N20; SC-816), PCF11 (ab134391), or HA epitope (a-HA; ab9110). After immunoprecipitation, protein G and protein A magnetic beads were added to the samples for 1 h at 4°C. After elution, RNA was extracted from the immunoprecipitation with hot-acid phenol. This was followed by reverse transcription (SuperScript III; ThermoFisher, 18080093) using an anchored oligo-dT primer.

*MYC*, *TBP*, and *SGK1* mRNA were detected and quantitated with RT-qPCR using gene-specific oligonucleotides.

### Microscopy

WNK1 localization images were taken on an OMX-V3 system (GE Healthcare) with a 1.42 NA 60× oil objective (Olympus), and SIM reconstruction was performed with SoftWorx software (Applied Precision). Different image channels were aligned using 200-nm TetraSpeck (Thermo Fisher Scientific) bead slides as a reference for the transformation. Images were analyzed and quantitated with FIJI software.

### RNA-seq

Raw RNA-seq data were adapter-trimmed with Cutadapt version 1.8.3 and mapped with TopHat version 2.0.13. Protein-coding genes were obtained from the ENSEMBL GRCh38.86 GTF file. Exons were extracted in R with the exonsBy function (package GenomicFeatures) and grouped by “gene.” Counts for each exonic feature were computed with summarizeOverlaps from the GenomicAlignments R package (parameters mode, “Union”; inter.feature, true; singleEnd, false; ignore.strand, false; fragments, true). Genes for which the sum of read counts through all eight samples was <50 were excluded. FPKM (fragments per kilobase per million mapped fragments) values (robust = TRUE) were computed with the DESeq2 FPKM function log_2_[FPKM(NU)/FPKM(CY)], and ratios for each experiment were computed for the heat map. To avoid low expression noise, only highly expressed genes with FPKM > 20 in the siLUC CY sample were considered and ordered according to the log_2_[(NU/CU)_siLUC_/(NU/CU)_siWNK1_] ratio.

The RNA-seq data are available in Gene Expression Omnibus under accession number GSE104982.

### Peptide affinity purification

Unmodified and phosphorylated peptides (see Supplemental Table S5) were coupled to SulfoLink coupling resin (ThermoFisher) according to the manufacturer's instructions. The coupled peptides were then incubated with HeLa nuclear extract and washed, and bound proteins were resolved on SDS-PAGE and assayed by either Western blot or MS. For MS, the samples were allowed to run only 5 mm into the gel. The whole gel slice was subjected to tryptic digestion.

### MS

Proteins were digested with trypsin overnight at 37°C. The resulting peptide mixtures were resuspended in 5% DMSO and 5% formic acid. They were separated on an Ultimate 3000 ultrahigh-performance liquid chromatography (UHPLC) system (Thermo Fischer Scientific) and electrosprayed directly into an QExactive mass spectrometer (Thermo Fischer Scientific) through an EASY-Spray nanoelectrospray ion source (Thermo Fischer Scientific). The peptides were trapped on a C18 PepMap100 precolumn (300-µm I.D. × 5 mm, 100 Å; Thermo Fisher Scientific) using solvent A (0.1% formic acid in water) at a pressure of 500 bars. The peptides were separated on an in-house-packed analytical column (75-µm I.D. packed with ReproSil-Pur 120 C18-AQ, 1.9 µm, 120 Å; Dr. Maisch GmbH) using a linear gradient of 15%–38% acetonitrile in 0.1% formic acid. The raw data were acquired on the mass spectrometer in a data-dependent mode (DDA). Full-scan MS spectra were acquired in the Orbitrap (scan range, 350–1500 *m/z*; resolution, 70,000; AGC target, 3e6, maximum injection time, 50 msec). After the MS scans, the 10 most intense peaks were selected for HCD fragmentation at 30% of normalized collision energy. HCD spectra were also acquired in the Orbitrap (resolution, 17,500; AGC target, 5e4; maximum injection time, 120 msec) with first fixed mass at 180 *m/z*. Charge exclusion was selected for unassigned and 1+ ions. The dynamic exclusion was set to 5–20 sec depending on the sample.

The data were searched against the United Protein Response *Homo sapiens* database (for samples purified from human cells) or a custom database containing the GST-PCF11 CID sequence as well as UniProt human and UniProt *E. coli* (for recombinant proteins) with the aid of the MASCOT (Matrix Science) search engine. For the peptide affinity purification binding partner study, semiquantitative label-free analysis (SINQ) was used as implemented in the Central Proteomics Facilities Pipeline (CPFP).

For data in Supplemental Table 2, experiments 1 and 2 were done in untreated cells, and experiment 3 was performed after 30 min of 50 nM calyculin A treatment to block phosphatase activity and boost phosphorylation levels.

### Computational tools

NLS predictions for the WNK1 protein were made using NLS Mapper (Kosugi et al. 2009) and NLStradamus (Nguyen Ba et al. 2009) on NCBI reference sequence NP_061852.2. The identified putative NLSs were AA563-572 (SLIKRKREQR; NLS Mapper and NLStradamus), AA1096-1106 (RTTKRHYRKSV; NLS Mapper and NLStradamus), and AA2121-2136 (SGRRRRPTKSKGSKSS; NLStradamus).

Kinase activity predictions on PCF11 were performed using GPS 2.1 (Xue et al. 2008) on NCBI reference sequence NP_056969.2. WNK1 family kinases had only one hit on T121 in the peptide SLFKLRSTWDEIFPL.

### PhosTag gel

The PhosTag compound (Wako Chemical Industries, AAL-107) was used to cast acrylamide gels according to the manufacturer's protocols.

### WNK activity inhibition

WNK463 compound was obtained from the Medical Research Council Protein Phosphorylation and Ubiquitylation Unit at the University of Dundee. Cells were treated with different concentrations of WNK463 inhibitor for 6 h as indicated. To test for inhibition of WNK1 activity, cells were subsequently stressed with the addition of 0.5 M sorbitol for 30 min to induce phosphorylation of OSR1 by WNK1.

## Supplementary Material

Supplemental Material
