# Supplementary material for: WNK1 kinase and the termination factor PCF11 connect nuclear mRNA export with transcription
Source: Genes Dev. 2017 Nov 1;31(21):2175–85. doi: 10.1101/gad.303677.117 (PMC5749165; doi:10.1101/gad.303677.117)
Supplement: Supplemental Material [file supp_31_21_2175__index.html]

WNK1 kinase and the termination factor PCF11 connect nuclear mRNA export with transcription — Supplemental Material 

# WNK1 kinase and the termination factor PCF11 connect nuclear mRNA export with transcription

## Supplemental Material

- Supplemental\_Data.pdf
- Supplemental\_Table\_3.xlsx
- Supplemental\_Table\_1.xlsx
- Supplemental\_Table\_4.xlsx
- Supplemental\_Table\_2.xlsx
- Supplemental\_Table\_5.xlsx
