# Supplementary material for: WNK1 kinase and the termination factor PCF11 connect nuclear mRNA export with transcription
Source: Genes Dev. 2017 Nov 1;31(21):2175–85. doi: 10.1101/gad.303677.117 (PMC5749165; doi:10.1101/gad.303677.117)
Supplement: Supplemental Material [file supp_gad.303677.117_Supplemental_Data.pdf]

Figure S1

(Related to Figure 1)

A.

Protein	Nb unique peptides	FDR
WNK1	11	0.001

B.

Peptides

Probability	Weight	Charge / Sequence
0.9996	0.99	ESFVDQVR
		- 2 ESFVDQVR
0.9996	0.99	FIVSPVPESR
		- 2 FIVSPVPESR
0.9996	0.99	IGDLGLATLK
		- 2 IGDLGLATLK
0.9996	1.00	SISNPPGSNLR
		- 2 SISNPPGSNLR
0.9996	1.00	SQQQDDIEELETK
		- 2 SQQQDDIEELETK
0.9995	1.00	EGPVLATSSGAGVFK
		- 2 EGPVLATSSGAGVFK
0.9993	1.00	VTSGVKPASFDK
		- 2 VTSGVKPASFDK
0.9988	0.99	ILNVSNKGDR
		- 2 ILNVSNKGDR
0.9988	1.00	VAIPEVK
		- 2 VAIPEVK
0.9982	1.00	QQVEQSSASQTGIK
		- 2 QQVEQSSASQTGIK
0.9943	1.00	EIQDLQSR
		- 2 EIQDLQSR

C.

sp|Q9H4A3-2|WNK1_HUMAN - Isoform 2 of Serine/threonine-protein kinase WNK1 OS=Homo sapiens GN=WNK1

Percent Coverage: 5.5%

```
MSGGAAEKQS STPGSLFLSP PAPAPKNGSS SDSSVGEKLG AAAADAVTGR TEEYRRRRHT MDKDSRGAAA TTTTTEHRFF
RRSVICDSNA TALELPGLPL SLPQPSIPAA VPQSAPPEPH REETVTATAT SQVAQQPPAA AAPGEQAVAG PAPSTVPSST
SKDRPVSQPS LVGSKEEPPP ARSGSGGGSA KEPQEERSQQ QDDIEELETK AVGMSNDGRF LKFDIEIGRG SFKTVYKGLD
TETTVEVAWC ELQDRKLTKS ERQRFKEEAE MLKGLQHPNI VRFYDSWEST VKGKKCIVLV TELMTSGTLK TYLKRFKVMK
IKVLRSWCRQ ILKGLQFLHT RTPPIIHRDL KCDNIFITGP TGSVKIGDLG LATLKRASFA KSVIGTPEFM APEMYEEKYD
ESVDVYAFGM CMLEMATSEY PYSECQNAAQ IYRRVTSGVK PASFDKVAIP EVKEIIEGCI RQNKDERYSI KDLLNHAFFQ
EETGVRVELA EEDDGEKIAI KLWLRIEDIK KLKGKYKDNE AIEFSFDLER DVPEDVAQEM VESGYVCEGD HKTMAKAIKD
RVSLIKRKRE QRQLVREEQE KKKQEESSLK QQVEQSSASQ TGIKQLPSAS TGIPTASTTS ASVSTQVEPE EPEADQHQQL
QYQQPSISVL SDGTVDSGQG SSVFTESRVS SQQTVSYGSQ HEQAHSTGTV PGHIPSTVQA QSQPHGVYPP SSVAQGQSQG
QPSSSSLTGV SSSQPIQHPQ QQGIQQTAPP QQTVQYSLSQ TSTSSEATTA QPVSQPQAPQ VLPQVSAGKQ STQGVSQVAP
AEPVAVAQTQ ATQPTTLASS VDSAHSDVAS GMSDGNENVP SSSGRHEGRT TKRHYRKSVR SRSRHEKTSR PKLRILNVSN
KGDRVVECQL ETHNRKMVTF KFDLDGDNPE EIATIMVNND FILAIERESF VDQVREIIEK ADEMLSEDVS VEPEGDQGLE
SLQGKDDYGF SGSQKLEGEF KQPIPASSMP QQIGIPTSSL TQVVHSAGRR FIVSPVPESR LRESKVFPSE ITDTVAASTA
QSPGMNLSHS ASSLSLQQAF SELRRAQMTE GPNTAPPNFS HTGPTFPVVP PFLSSIAGVP TTAAATAPVP ATSSPPNDIS
TSVIQSEVTV PTEEGIAGVA TSTGVVTSGG LPIPPVSESP VLSSVVSSIT IPAVVSISTT SPSLQVPTST SEIVVSSTAL
YPSVTVSATS ASAGGSTATP GPKPPAVVSQ QAAGSTTVGA TLTSVSTTTS FPSTASQLCI QLSSSTSTPT LAETVVVSAH
SLDKTSHSST TGLAFSLSAP SSSSSPGAGV SSYISQPGGL HPLVIPSVIA STPILPQAAG PTSTPLLPQV PSIPPLVQPV
ANVPAVQQTL IHSQPQPALL PNQPHTHCPE VDSDTQPKAP GIDDIKTLEE KLRSLFSEHS SSGAQHASVS LETSLVIEST
VTPGIPTTAV APSKLLTSTT STCLPPTNLP LGTVALPVTP VVTPGQVSTP VSTTTSGVKP GTAPSKPPLT KAPVLPVGTE
LPAGTLPSEQ LPPFPGPSLT QSQQPLEDLD AQLRRTLSPE MITVTSAVGP VSMAAPTAIT EAGTQPQKGV SQVKEGPVLA
TSSGAGVFKM GRFQVSVAAD GAQKEGKNKS EDAKSVHFES STSESSVLSS SSPESTLVKP EPNGITIPGI SSDVPESAHK
TTASEAKSDT GQPTKVGRFQ VTTTANKVGR FSVSKTEDKI TDTKKEGPVA SPPFMDLEQA VLPAVIPKKE KPELSEPSHL
NGPSSDPEAA FLSRDVDDGS GSPHSPHQLS SKSLPSQNLS QSLSNSFNSS YMSSDNESDI EDEDLKLELR RLRDKHLKEI
QDLQSRQKHE IESLYTKLGK VPPAVIIPPA APLSGRRRRP TKSKGSKSSR SSSLGNKSPQ LSGNLSGQSA ASVLHPQQTL
HPPGNIPESG QNQLLQPLKP SPSSDNLYSA FTSDGAISVP SLSAPGQGTS STNTVGATVN SQAAQAQPPA MTSSRKGTFT
DDLHKLVDNW ARDAMNLSGR RGSKGHMNYE GPGMARKFSA PGQLCISMTS NLGGSAPISA ASATSLGHFT KSMCPPQQYG
FPATPFGAQW SGTGGPAPQP LGQFQPVGTA SLQNFNISNL QKSISNPPGS NLRTT
```

D.

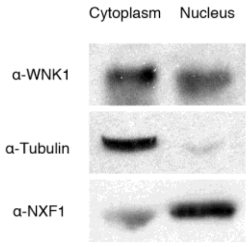

(A-C) Mass Spectrometric evidence for WNK1-PCF11 interaction. PCF11 IP was performed in nuclear extract and co-immunoprecipitated interacting proteins analyzed by MS. The only kinase found in the experiment was WNK1. **A.** Summary of detected peptides. FDR is derived from comparison with control mock IP experiment (CPFP SINQ). **B.** Identified WNK1 peptides. **C.** Coverage of the identified WNK1 peptides both at N- and C-terminus of the sequence indicates likely presence of full-length protein in the IP. **D.** Western blot for the detection of WNK1 in cytoplasmic and nuclear HeLa cell extracts. Tubulin and the export factor NXF1 were used as controls for fractionation.

Figure S2

(Related to Figure 1)

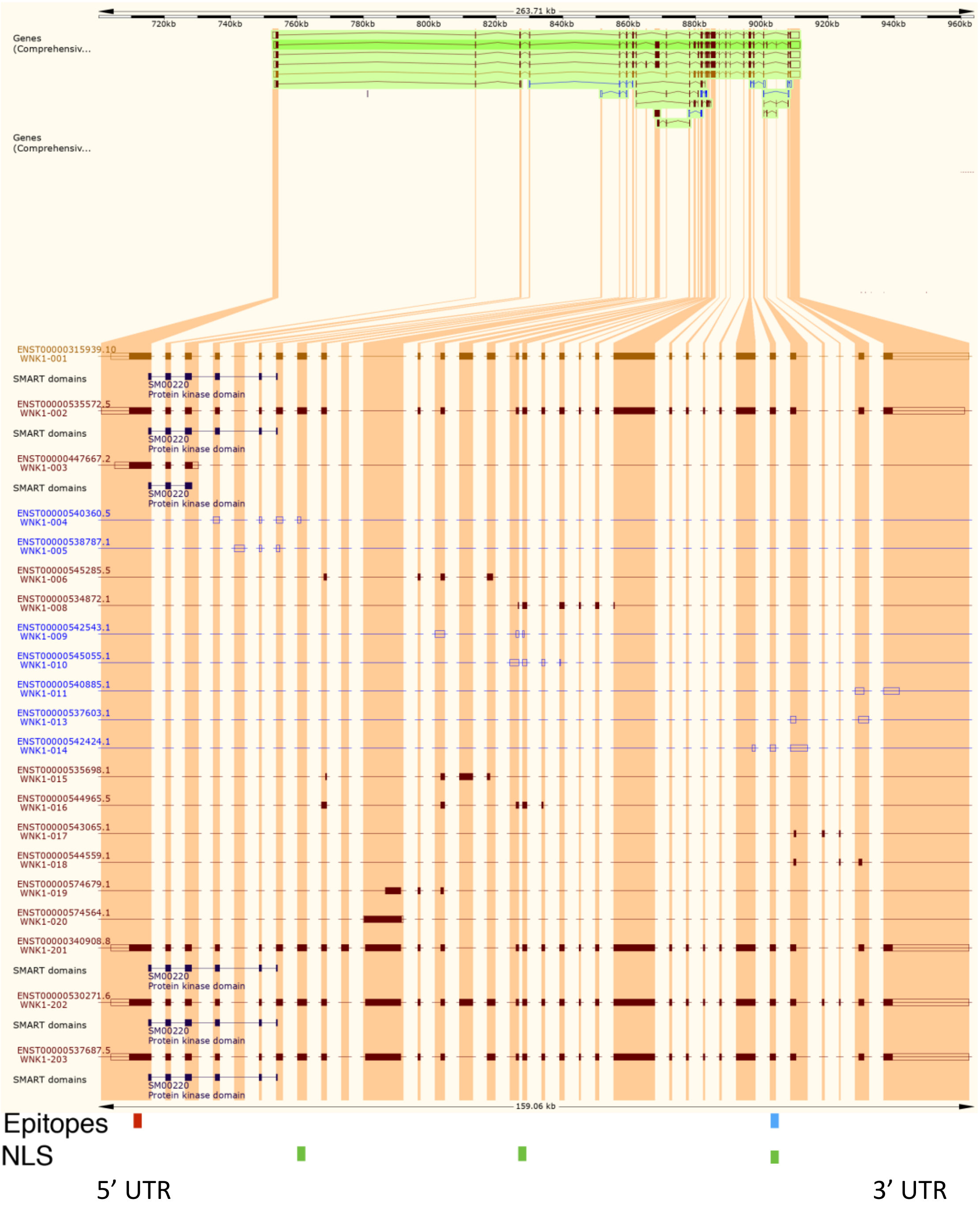

Splicing isoforms of *WNK1*. 6 of the 21 total annotated isoforms contain the catalytic kinase domain. Red box represents the recognition epitope of the antibody used in previous studies (Tu et al. 2011), a blue box represents the epitope of the antibody used in this study (Bethyl, A301-514). NLS stands for Nuclear Localization Signal (see also Material and Methods) and is shown in green.

Figure S3

(Related to Figures 1 and 2)

A

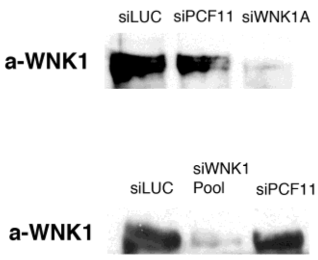

B

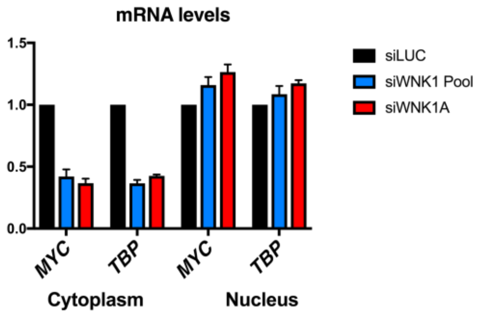

C

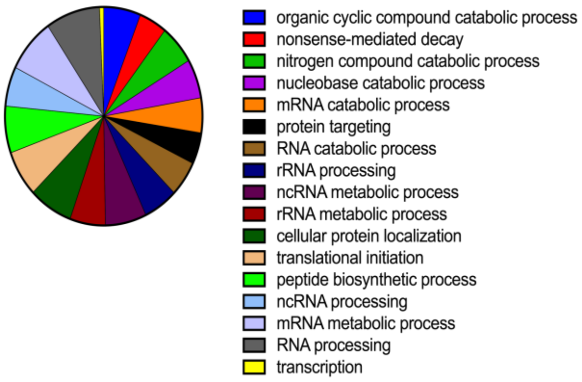

A. Detection of WNK1 depletion levels upon siRNA treatment. Two different siRNAs where used: siWNK1A (single siRNA targeting) and siWNK1 Pool (OnTARGET Dharmakon siRNA Smart pool). Western blot with a-WNK1 antibody (Bethyl, A301-515A). siLUC and siPCF11 siRNA were used as control. **B.** Effect of depletion of WNK1 with different siRNAs on mRNA export. Measurement of nuclear and cytoplasmic mRNA levels of *TBP* and *MYC* genes upon WNK1 depletion (Error bars represent standard error). **C.** GO-analysis of the genes with affected mRNA export upon WNK1 depletion. Details for GO analysis in Supplemental Table 1. No GO categories showed significant enrichment.

Figure S4

(Related to Figure 3)

A

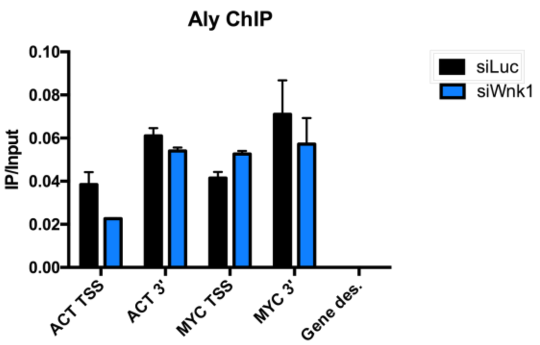

B

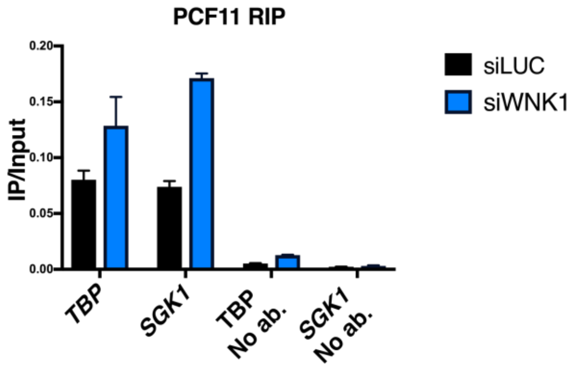

A. ChIP experiment for Aly recruitment. siRNA mediated depletion of WNK1 does not have a significant effect on Aly recruitment on the Actin and *MYC* genes (Summary data of three technical repeats, error bars represent standard error). **B.** Levels of *TBP* and *SGK1* mRNA associated with immunoprecipitated PCF11 for DNase treated extracts of control and WNK1 depleted cells (Summary data of three technical repeats of, error bars represent standard error).

Figure S5

(Related to Figure 4)

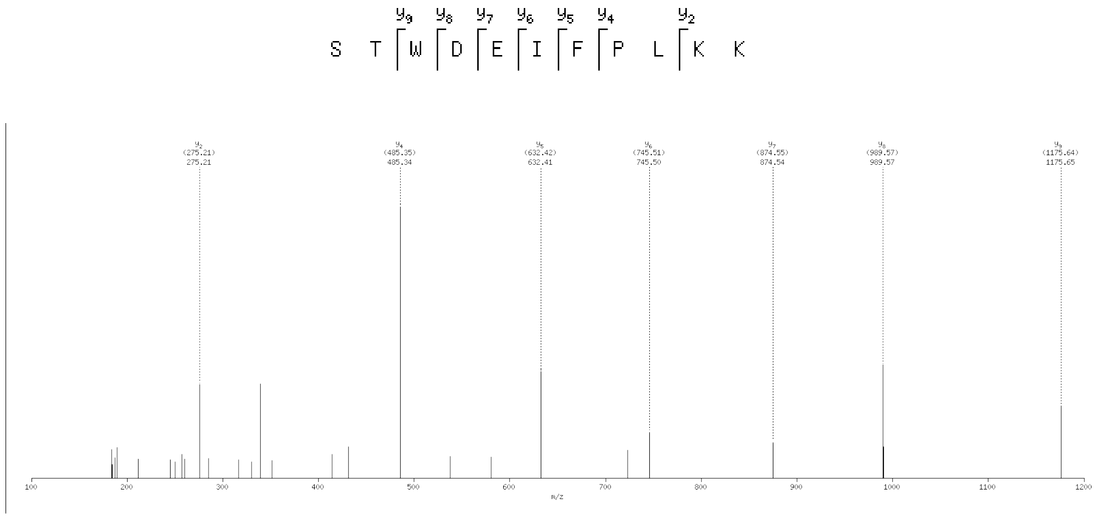

Monoisotopic mass of neutral peptide Mr(calc): 1442.6847
Ions score: 62 Expect: 1.5e-06
Matches: 7/1184 fragment ions using 9 most intense peaks

#	Immon.	a	a++	b	b++	b*	b*++	b0	b0++	Seq.	y	y++	y*	y*++	y0	y0++	#
1	60.0444	60.0444	30.5258	88.0393	44.5233			70.0287	35.5180	S							11
2	154.0264	241.0584	121.0328	269.0533	135.0303			251.0427	126.0250	T	1356.6599	678.8336	1339.6334	670.3203	1338.6494	669.8283	10
3	159.0917	427.1377	214.0725	455.1326	228.0700			437.1221	219.0647	W	***1175.6459***	588.3266	1158.6194	579.8133	1157.6354	579.3213	9
4	88.0393	542.1647	271.5860	570.1596	285.5834			552.1490	276.5781	D	***989.5666***	495.2869	972.5401	486.7737	971.5560	486.2817	8
5	102.0550	671.2072	336.1073	699.2022	350.1047			681.1916	341.0994	E	***874.5397***	437.7735	857.5131	429.2602	856.5291	428.7682	7
6	86.0964	784.2913	392.6493	812.2862	406.6468			794.2757	397.6415	I	***745.4971***	373.2522	728.4705	364.7389			6
7	120.0808	931.3597	466.1835	959.3546	480.1810			941.3441	471.1757	F	***632.4130***	316.7101	615.3865	308.1969			5
8	70.0651	1028.4125	514.7099	1056.4074	528.7073			1038.3968	519.7021	P	***485.3446***	243.1759	468.3180	234.6627			4
9	86.0964	1141.4966	571.2519	1169.4915	585.2494			1151.4809	576.2441	L	388.2918	194.6496	371.2653	186.1363			3
10	101.1073	1269.5915	635.2994	1297.5864	649.2969	1280.5599	640.7836	1279.5759	640.2916	K	***275.2078***	138.1075	258.1812	129.5942			2
11	101.1073									K	147.1128	74.0600	130.0863	65.5468			1

All matches to this query

Score	Mr(calc)	Delta	Sequence	Site Analysis
62.1	1442.6847	0.0008	STWDEIFPLKK	Phospho T2 50.00%
62.1	1442.6847	0.0008	STWDEIFPLKK	Phospho S1 50.00%

Mass spectrometry evidence of *in vivo* PCF11 CID phosphorylation. PCF11 was immunoprecipitated from nuclear extract, resolved on SDS-PAGE, the coomassie band corresponding to the PCF11 protein cut out, digested with trypsin and subject to Mass Spectrometry. Shown are the peptide spectra and fragment ions of the CID phosphorylated peptide from MASCOT. Three independent biological repeats showed evidence of phosphorylated S120/T121 peptides (Supplemental Table 2). Note that we were unable to unambiguously determine which of the neighbouring S120/T121 residues is phosphorylated *in vivo*.

Figure S6

(Related to Figure 4)

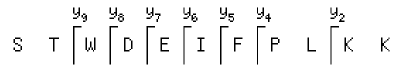

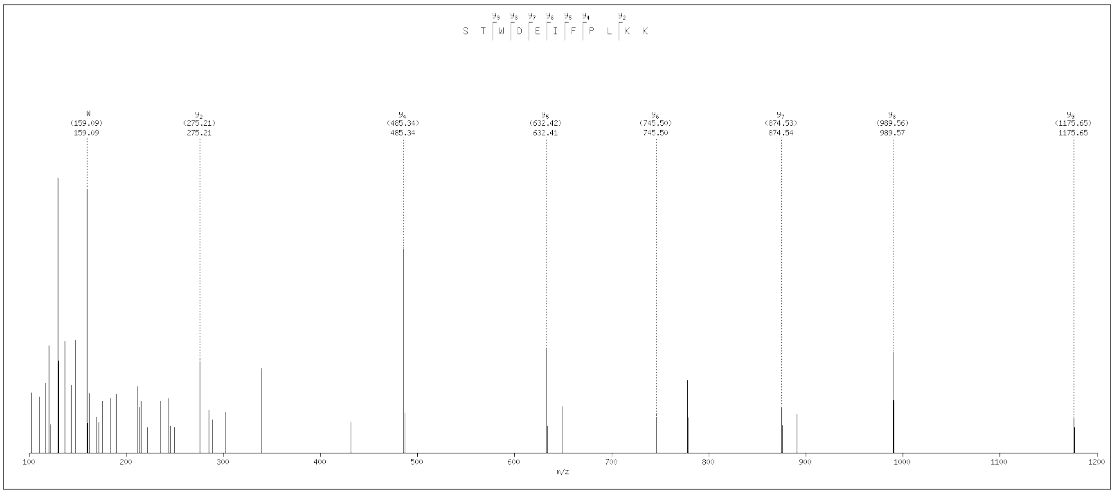

Monoisotopic mass of neutral peptide Mr(calc): 1442.6847
Ions Score: 32 **Expect:** 0.0011
Matches : 8/184 fragment ions using 36 most intense peaks

#	Immon.	a	a++	b	b++	b*	b*++	b0	b0++	Seq.	y	y++	y*	y*++	y0	y0++	#
1	60.0444	60.0444	30.5258	88.0393	44.5233			70.0287	35.5180	S							11
2	154.0264	241.0584	121.0328	269.0533	135.0303			251.0427	126.0250	T	1356.6599	678.8336	1339.6334	670.3203	1338.6494	669.8283	10
3	159.0917	427.1377	214.0725	455.1326	228.0700			437.1221	219.0647	W	***1175.6459***	588.3266	1158.6194	579.8133	1157.6354	579.3213	9
4	88.0393	542.1647	271.5860	570.1596	285.5834			552.1490	276.5781	D	***989.5666***	495.2869	972.5401	486.7737	971.5560	486.2817	8
5	102.0550	671.2072	336.1073	699.2022	350.1047			681.1916	341.0994	E	***874.5397***	437.7735	857.5131	429.2602	856.5291	428.7682	7
6	86.0964	784.2913	392.6493	812.2862	406.6468			794.2757	397.6415	I	***745.4971***	373.2522	728.4705	364.7389			6
7	120.0808	931.3597	466.1835	959.3546	480.1810			941.3441	471.1757	F	***632.4130***	316.7101	615.3865	308.1969			5
8	70.0651	1028.4125	514.7099	1056.4074	528.7073			1038.3968	519.7021	P	***485.3446***	243.1759	468.3180	234.6627			4
9	86.0964	1141.4966	571.2519	1169.4915	585.2494			1151.4809	576.2441	L	388.2918	194.6496	371.2653	186.1363			3
10	101.1073	1269.5915	635.2994	1297.5864	649.2969	1280.5599	640.7836	1279.5759	640.2916	K	***275.2078***	138.1075	258.1812	129.5942			2
11	101.1073									K	147.1128	74.0600	130.0863	65.5468			1

All matches to this query

Score	Mr(calc)	Delta	Sequence	Site Analysis
31.5	1442.6847	-0.0127	STWDEIFPLKK	Phospho T2 50.00%
31.5	1442.6847	-0.0127	STWDEIFPLKK	Phospho S1 50.00%

Mass spectrometry analysis of recombinant WNK1 kinase domain activity on recombinant GST-PCF11-CID *in vitro*. GST-PCF11-CID was incubated with WNK1 as described, resolved on SDS-PAGE, the coomassie band corresponding to GST-PCF11-CID cut out, digested with trypsin and subject to Mass Spectrometry. Shown are the peptide spectra and fragment ions of the CID phosphorylated peptide from MASCOT. Note that we were unable to unambigously determine which of the neighbouring S120/T121 residues is phosphorylated. All identified peptides for GST-PCF11-CID are shown in Supplemental Table 3.

Figure S7

(Related to Figure 5)

A

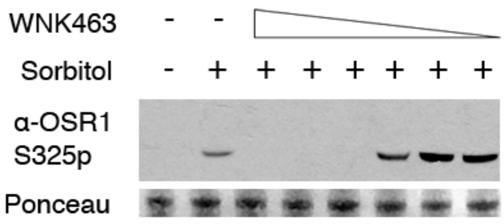

B

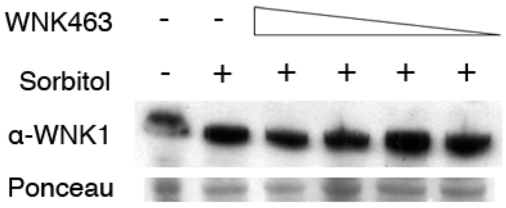

C

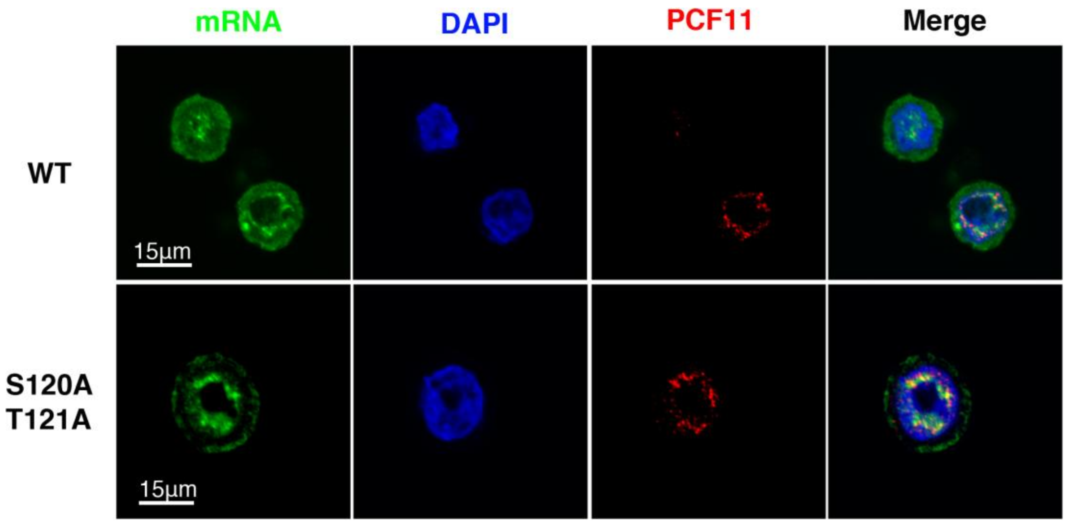

A. Western blot for the detection of OSR1 S325 phosphorylation by WNK1 upon inhibition of WNK kinase domain. Cells were incubated with the WNK463 inhibitor for 6 hrs, followed by 30 min treatment with 0.5M Sorbitol to induce OSR1 S325 phosphorylation by WNK1. A range of concentrations from 1mM to 100nM was tested. **B.** Detection of WNK1 levels upon its inhibition with WNK463. Cells were incubated with WNK463 for 6 hrs, followed by 30min treatment with 0.5M Sorbitol, same concentrations as panel A were tested. **C.** Fluorescent oligo-dT FISH for the detection of mRNA levels in cells expressing WT or S120A/T121A PCF11. Alexa Fluor 488-dT_{23} was used as a probe and DAPI for DNA staining. α-HA antibody was used for staining of ectopically expressed PCF11.

Figure S8

(Related to Figure 5)

A.

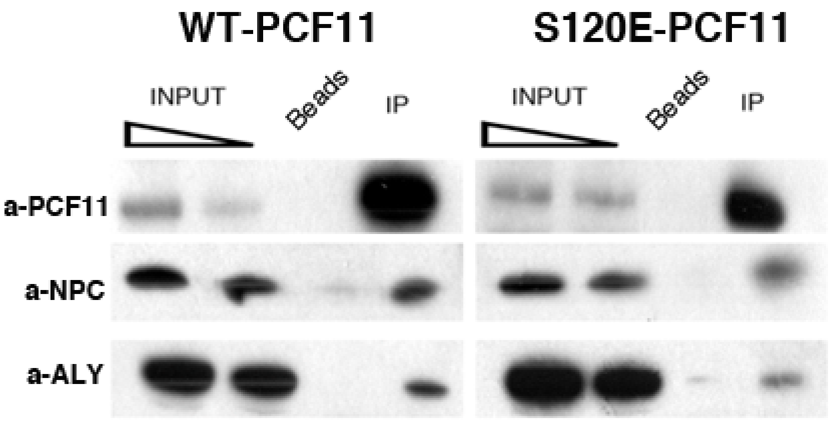

B.

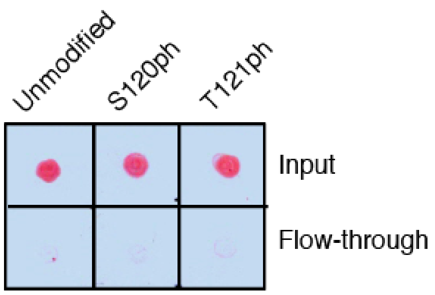

A.Co-IP experiments of ectopically expressed PCF11. WT PCF11 co-immunoprecipitates with the nuclear pore complex (NPC) and the export factor ALY. Expression of the phosphomimetic PCF11 does not show a significant change in this interaction **B.** Peptide coupling efficiency of S120/T121 unmodified, as well as phosphorylated S120ph and T121ph peptides (sequences available in Supplemental table 5). Peptide concentration was monitored by ponceau red staining on a dot blot before (input) and after coupling of the peptides to sulfolink beads (flow-through). All peptides coupled very efficiently to the resin as evidenced by only trace quantities detectable in the flow-through.

Supplemental tables list

Supplemental table 1

GO analysis of genes whose export is affected by WNK1 depletion.

Supplemental table 2

In vivo PCF11 phosphorylation sites detected in Mass Spectrometry in HeLa cells (3 experiments).

Supplemental table 3

Phosphorylation sites on GST-PCF11CID detected by Mass Spectrometry after in vitro kinase assay with WNK1 kinase domain.

Supplemental table 4

Mass Spectrometry analysis of proteins bound in peptide affinity purification using PCF11 CID peptides. Shown are Spectral index (MIC SIn) and ratios of protein abundance between phosphorylated and unphosphorylated peptide used as bait obtained through CPFP.

Supplemental table 5

Sequences of oligonucleotides and peptides used in this study.
